# Global Incidence of IgA Nephropathy by Race and Ethnicity: A Systematic Review

**DOI:** 10.34067/KID.0000000000000165

**Published:** 2023-05-25

**Authors:** Krzysztof Kiryluk, Daniel E. Freedberg, Jai Radhakrishnan, Leslie Segall, Judith S. Jacobson, Mohit Mathur, Sumit Mohan, Alfred I. Neugut

**Affiliations:** 1 Division of Nephrology, Vagelos College of Physicians and Surgeons, Columbia University, New York, New York; 2 Division of Digestive and Liver Diseases, Vagelos College of Physicians and Surgeons, Columbia University, New York, New York; 3 Department of Epidemiology, Mailman School of Public Health, Columbia University, New York, New York; 4 Visterra, Inc., Waltham, Massachusetts; 5 Division of Hematology/Oncology, Vagelos College of Physicians and Surgeons, Columbia University, New York, New York

**Keywords:** chronic glomerulonephritis, epidemiology and outcomes, IgA nephropathy, minority health and disparities

## Abstract

**Key Points:**

- In 16 studies conducted abroad, IgA nephropathy incidence varied from 0.06 in South Africa to 4.2 per 100,000 in Japan.
- Globally, the incidence of IgA nephropathy seemed higher in Asians than in non-Asians and higher in male patients than in female patients.
- Five studies conducted in the United States found no consistent difference in incidence between Black patients and White patients.

**Background:**

The reported incidence of IgA nephropathy varies widely across studies and may vary on the basis of race/ethnicity. This study systematically reviewed the incidence of IgA nephropathy in the United States and other countries and explored variability on the basis of the racial/ethnic composition and other demographic characteristics of different populations.

**Methods:**

This was a systematic review. Studies were eligible for inclusion if they contained data collected from January 1, 1974, to December 31, 2021, and reported IgA nephropathy incidence at a population level (*i.e.*, cases of IgA nephropathy per 100,000 population).

**Results:**

Five US and 16 international studies were included; three of the US studies reported the race-specific incidence of IgA nephropathy. In the United States, the reported incidence of IgA nephropathy ranged from 0.39 per 100,000 in Tennessee to 1.4 per 100,000 in Minnesota; internationally, IgA nephropathy ranged from 0.06 per 100,000 in South Africa to 4.2 per 100,000 in Japan. Findings regarding the incidence of IgA nephropathy in the United States by race were inconsistent: One study found a higher incidence among White patients compared with Black patients, one study found a lower incidence in White patients, and one study found no difference. Globally, the incidence of IgA nephropathy seemed to be higher in Asian than in non-Asian populations and higher in male patients than in female patients.

**Conclusions:**

Reported incidence of IgA nephropathy varies widely; there is no consensus regarding the relationship between race and IgA nephropathy. Incidence rates seemed to be higher in Asians than non-Asians and in male patients than female patients. We recommend that future studies should report IgA nephropathy incidence rates by race/ethnicity and account for the demographic characteristics of the background population.

## Graphical abstract

**Figure:**
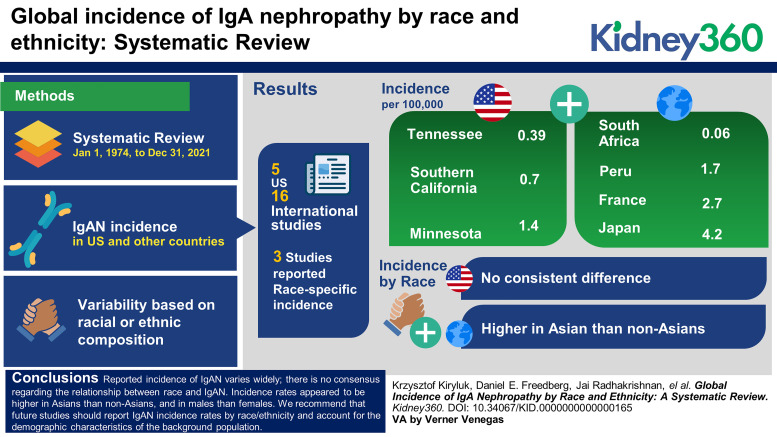

## Introduction

IgA nephropathy is diagnosed on the basis of the finding of dominant or codominant IgA deposition in the glomeruli by immunofluorescence microscopy. Failure to biopsy—because of either lack of health care access or rapid progression to ESKD—may result in underdiagnosis. Previous studies have suggested that higher rates of biopsy among those with kidney injury and suspected IgA nephropathy are associated with higher local estimates of IgA nephropathy.^1^ Race/ethnicity may affect IgA nephropathy incidence estimates both because genetic factors could contribute to true race-based risk for IgA nephropathy and because race may also be associated with access to kidney biopsy. In this review, we sought to estimate the incidence of IgA nephropathy in US populations, place these estimates within an international context, and relate them to race and other demographic characteristics.

IgA nephropathy incidence has been reported to be higher among East Asians than among other groups.^2–7^ Population-based genome-wide association studies have identified multiple IgA nephropathy susceptibility loci for sporadic IgA nephropathy, but full understanding of the genetics of IgA nephropathy has not been reached.^8^ International studies have typically stated that the incidence of IgA nephropathy is highest in populations of Asian descent, lower in populations of European descent, and lower still in populations of African descent.^9,10^ However, Europeans are over-represented in most studies, and research on IgA nephropathy is sparse in African populations where lack of access to biopsy may lead to underreporting of IgA nephropathy.^11,12^ Meanwhile, relatively little is known regarding the incidence of IgA nephropathy in South Americans or among US Hispanics, who were 18.7% of the US population in 2020.^13,14^

Most studies of IgA nephropathy incidence have been conducted in racially homogenous countries, making interstudy comparisons perilous. We systematically reviewed the relevant global literature, seeking to understand the roles of race/ethnicity and other factors in IgA nephropathy incidence in the United States and elsewhere.

## Methods

### Selection of Regions

Initially, we focused on the few studies of the demographic characteristics associated with IgA nephropathy within the United States. For global context, we also examined published IgA nephropathy incidence data from all continents and major world regions: North America, Europe, Asia, South America, Australia, and Africa.

### Selection of Studies

Studies were eligible for inclusion if they contained data collected from 1974 to 2021, had abstracts and articles written in English, and reported IgA nephropathy incidence at a population level. L. Segall conducted the initial literature search. A.I. Neugut and D.E. Freedberg screened the abstracts. When there was lack of agreement, complete articles were reviewed, and ties were resolved by consensus. Studies were excluded if they did not directly report IgA nephropathy incidence and did not provide sufficient data to allow it to be calculated (*i.e.*, the annual number of IgA cases or the size of the at-risk population could not be determined). We included reports of regional or population-based IgA nephropathy incidence so that we could compare locations with different demographic makeups. When studies did not directly report IgA nephropathy incidence rates, we calculated them by dividing the number of IgA nephropathy cases reported in the study by the at-risk population. We included all US studies that met the entry criteria and up to two studies each for non-US nations; when we found more than two studies of a non-US nation, we selected the two most recent ones. Relevant studies were identified by searching PubMed, Embase, Google, and Google Scholar using the terms “IgA nephropathy incidence,” “IgAN prevalence,” “IgAN epidemiology,” and “IgAN incidence by race.” We also searched for relevant studies using individual country names to ensure inclusion of countries from all the intended regions.

### Data Extraction

From the studies identified, we extracted bibliographic information, IgA nephropathy incidence data, and, if reported, country-specific data on the demographic makeup and size of the background population. When a country-specific study did not report such data, we used additional sources, such as national registry data, to determine the country's population and demographic characteristics as of the year closest to the last reported year of the study.

### IgA Nephropathy Incidence and Other Study Measures

We reported IgA nephropathy incidence measured per 100,000 person-years to compare countries and regions, and we standardized study data to this metric when necessary. Additional study measures included the time range of data collection, population age, sex data, and study design.

### Classification of Race

In US studies, we categorized race/ethnicity on the basis of the US census categories and data provided by individual studies. The categories were White, non-Hispanic; Black, non-Hispanic; Hispanic; Native American and Alaskan Native; Asian; Native Hawaiian and Other Pacific Islander; two or more races; and other (the US census provides the other category for respondents who do not identify with any of the above listed categories). It was unknown whether race/ethnicity was classified on the basis of self-identification or using alternative methods.^15^ Race and ethnicity data were not extracted from international studies.

### Statistical Approach

We developed descriptive statistics including calculations of medians and interquartile ranges, developed graphs to visualize the data, and analyzed correlations where appropriate. Because we found only a relatively small number of studies, we elected not to perform tests of statistical significance. This study was deemed exempt by the Columbia University Institutional Review Board.

## Results

### Studies Identified

**Figure 1.**
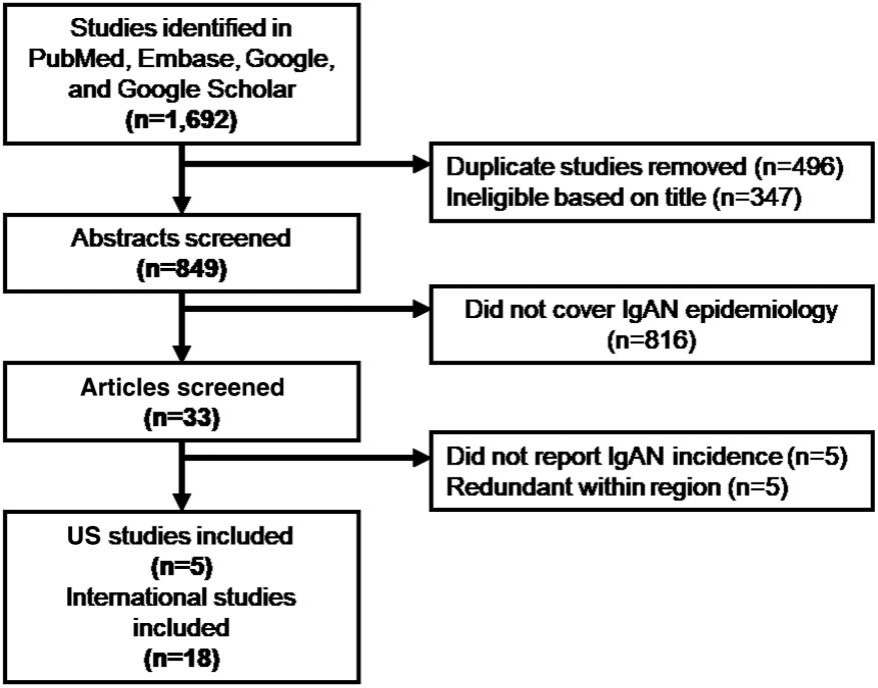
Flow diagram of studies reviewed.

Our search terms initially identified 1692 studies, of which 33 seemed to meet criteria for inclusion on the basis of the study abstracts (*i.e.*, these studies seemed to report a population-based incidence for IgA nephropathy). We reviewed these 33 studies in full and excluded ten, keeping five US and 18 international studies for data extraction (Figure 1). The US studies were conducted in Central and Eastern Kentucky; Olmsted County, Minnesota; Shelby County, Tennessee; New Mexico; and Southern California. The international studies that met the criteria for inclusion were conducted in France, the United Kingdom, Northern Ireland, the Netherlands, Germany, the Czech Republic, Estonia, Japan, Singapore, Australia, Peru, Brazil, and South Africa.

### Incidence of IgA Nephropathy in US Studies

**Figure 2.**
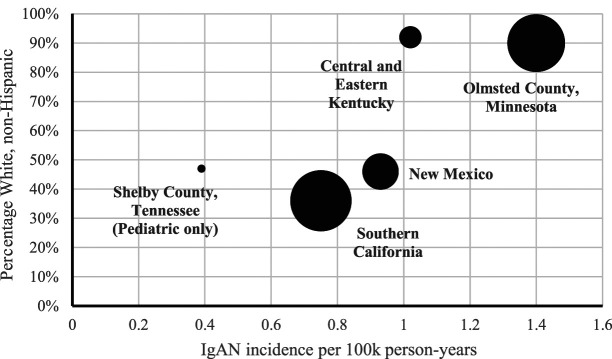
**Relationship between proportion of White patients in the background population and incidence of IgA nephropathy, in the five US incidence studies.** The size of the marker reflects the size of the background population of each study.

In the five US studies, the overall incidence of IgA nephropathy ranged from 0.39 (Tennessee) to 1.4 (Minnesota) per 100,000 population United States (Table 1).^9,13^ Three studies reported incidence by race. Two reported a higher incidence in individuals self-identified as White compared with Black (Kentucky and Southern California), whereas the study from Tennessee reported a higher incidence in Black patients.^16–18^ Next, we sought to understand the relationship between race and IgA nephropathy incidence by plotting the overall IgA nephropathy incidence against the proportion of individuals self-identified as White non-Hispanics within the study population (Figure 2). We observed a quasilinear relationship, although with only five data points.

**Table 1.** The incidence of IgA nephropathy reported in US studies

Study Details and Overall Incidence of IgA Nephropathy				Incidence of IgA Nephropathy Per 100,000, Stratified by Race					
Study Location	Time of Data Collection	Overall Incidence of IgA Nephropathy (Per 100,000)	Incidence of IgA Nephropathy by Time Period (Per 100,000)	White^a^	Black^a^	Hispanic	Asian^a^	Other^a^	Male (%)
Shelby County, Tennessee	1975–1994	0.39	Not reported	0.3	0.57	Not reported	Not reported	Not reported	77%
Central and Eastern Kentucky	1975–1994	1975–1984: 0.62 1985–1994: 1.02	1975–1979: 0.54 1980–1984: 0.71 1985–1989: 0.8 1990–1994: 1.24	1.07	1.02	Not reported	Not reported	Not reported	59%
Olmsted County, Minnesota	1974–2003	1.4	1974–1983: 0.7 1984–1993: 0.9 1994–2003: 2.1	Not reported					Not reported
New Mexico (entire state)	2000–2005	0.93	Not reported	Not reported					51%
Southern California	2000–2011	0.7	2000: 0.1 2001: 0.3 2002: 0.2 2003: 0.2 2004: 0.2 2005: 0.2 2006: 0.4 2007: 1.4 2008: 1.1 2009: 1.2 2010: 1.6 2011: 1.6	0.72	0.1	0.96	2.75	0.13	57%

a Non-Hispanic.

### Racial Background in US Studies

**Figure 3.**
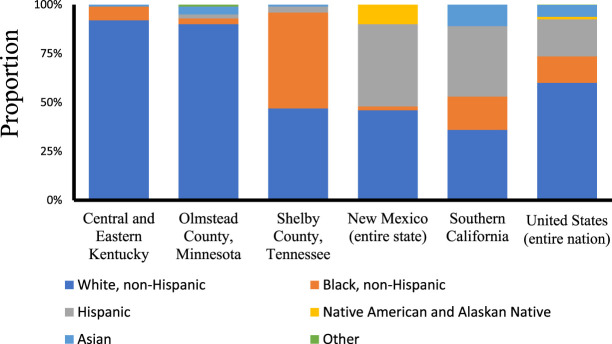
**Racial/ethnic composition of US locations, taken from the five US studies.** Data not reported in the study were collected from census reports collected at the end of the study periods. The composition of the entire US population is shown for comparative purposes.

To contextualize our data, we visualized the racial background of each US region where IgA nephropathy incidence was studied (Figure 3 and Table 2). The regions differed in racial/ethnic composition. The populations of Kentucky and Minnesota were 90% and 92% White, respectively, whereas Tennessee, New Mexico, and Southern California were more diverse.^7, 17–19^ Tennessee reported the highest proportion Black population (49%) and New Mexico the highest proportion Hispanic population (42%).^18^ The five US studies are reviewed in detail below.

**Table 2.** Demographic characteristics of the US populations studied in relation to the incidence of IgA nephropathy

Study and Population Details				Racial/Ethnic Compositions of Background Populations							
Study Location	Reference Population Size	Age Ranges Included	Median Ages	White, %	Black, %	Hispanic, %	Asian, %	Native American and Alaskan Native	Native Hawaiian and Pacific Islander	Two or More Races	Other, %
Shelby County, Tennessee	200,000	0–18	Not reported	47	49	3	2	0.20%	0.04%	1%	1
Central and Eastern Kentucky^a^	1 million	9 to >70	Not reported	92	7	0.01	1	<1%	<1%	<1%	1
Olmsted County, Minnesota	3 million	9 to ≥80	Not reported	90	3	2	4	0.26%	0.03%	2%	0.92
New Mexico (entire state)	1·8 million	5–86	33	46	2	42	1	10%	0.10%	<1%	0.60
Southern California	>3 million	18–84	42	36	17	36	12	<1%	<1%	<1%	3

a Forty-five counties including Fayette County in Central and Eastern Kentucky. The background population data are drawn from the 1990 census for all of Kentucky.

### Incidence of IgA Nephropathy in Southern California

The study conducted in Southern California was performed within the Kaiser Permanente health care system from January 2000 through December 2011 in a background population of over three million.^17^ Kaiser Permanente is a prepaid employment-based health plan with a large membership that reflects the underlying relatively diverse population of insured Southern Californians. The overall incidence of IgA nephropathy was 0.7 per 100,000. The highest incidence by race was 2.75 per 100,000 among Asians, and the lowest was 0.1 per 100,000 among Black patients.^17^

### Incidence of IgA Nephropathy in New Mexico

The study conducted in New Mexico examined >98% of kidney biopsies performed in the state of New Mexico between 2000 and 2005 in a background population of 1.8 million.^7^ The overall incidence of IgA nephropathy was 0.93 per 100,000. The biopsied population was 21% non-Hispanic White, 49% Hispanic, 21% American Indian, and 9% other (African American, Asian/Pacific Islander, and of unknown ethnicity), but the incidence of IgA nephropathy was not reported in a race-specific manner. In comparison, American Indians were 10% of the New Mexico population at the time and 2% of the overall US population.^20^ Non-Hispanic White patients were 21% of the cases and 45% of the New Mexico population but 67% of the overall US population in 2005.^21^

### Incidence of IgA Nephropathy in Olmsted County, MN

The study conducted in Olmsted County estimated the age-adjusted incidence of IgA nephropathy over three decades (1974–2003) in a background population of three million in a Mayo Clinic dataset.^19^ These data were assembled through multiple studies conducted within the Rochester Epidemiology Project and the Southeast Minnesota Birth Cohort.^22^ The overall IgA nephropathy incidence was 1.4 cases per 100,000 person-years. The incidence of IgA nephropathy was not reported by race, but Olmsted County has limited racial and socioeconomic diversity; it was 90% White at the end of the study period.

### Incidence of IgA Nephropathy in Central and Eastern Kentucky

The study conducted in Central and Eastern Kentucky estimated the incidence of IgA nephropathy between 1975 and 1994 in a background population of one million.^16^ The study included all biopsies taken in this time in 45 Kentucky counties, which included a few populous counties such as Fayette and Pike but was primarily composed of rural counties. The overall incidence of IgA nephropathy varied from 0.62 to 1.02 per 100,000 during the years of the study. Incidence was separately reported for White patients (1.07 per 100,000) and for Black patients (1.02 per 100,000), but not for other races or ethnicities. The background population in these counties at the time was 92% White. The authors stated that although familial IgA nephropathy had been identified in a population in Kentucky previously, their estimates reflected nonfamilial IgA nephropathy incidence in Central and Eastern Kentucky.^3^

### Incidence of IgA Nephropathy in Tennessee

The Shelby County, Tennessee study provided data on race and incidence of IgA nephropathy in children from 1975 to 1994 in a background population of 200,000.^18^ On the basis of only 17 cases, incidence was 0.39 per 100,000 overall, 0.3 per 100,000 for White patients, and 0.57 per 100,000 for Black patients. Incidence was not reported for other races or ethnicities. The background population in Shelby County at the time of the study was 47% White, 49% Black, and 7% other (*e.g.*, more than one race).

### Incidence of IgA Nephropathy in the United States in the Context of International Studies

**Figure 4.**
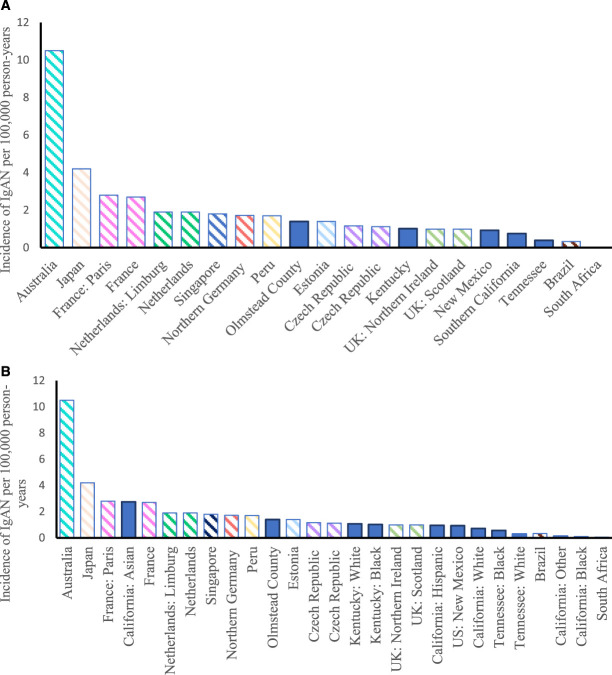
**Variability of IgA nephropathy across populations internationally and in the US, as described in the text.** (A) Incidence of IgA nephropathy in US and international studies. (B) Incidence of IgA nephropathy in US and international studies, with US incidence data presented separately on the basis of race.

The overall incidence of IgA nephropathy internationally ranged from a high of 10.5 per 100,000 in Australia to a low of 0.06 per 100,000 in South Africa (Figure 4A and Table 3)^9,23^; the median was 1.4 per 100,000 (interquartile range, 0.96–1.9), which was equal to or higher than the incidence in any US study.^19^ Figure 4B shows incidence data by race for the three US studies, in the context of the international studies.^16–18^.

**Table 3.** Incidence of IgA nephropathy among the included international studies

Study Location	Overall Incidence (Per 100,000)	Time of Data Collection	Reference Population Size	Ages Included, yr	Mean Age, yr	Male (%)
**Western Europe**						
Paris, France	2.8	1994–2001	2 million	>18	37	Not reported
France	2.7	1976–1990	400,000	10–80	35–55 (over time)	73%
United Kingdom—Scotland	0.99	2000–2014	1.5 million	16–65	56.4	Not reported
Northern Ireland	0.99	1976–2005	1.7 million	16–92	49	80%
The Netherlands	1.9	1979–1985	1.5 million	16–65	Not reported	Not reported
Limburg, the Netherlands	1.9	1985–2003	630k	Not reported	41	74%
Northern Germany	1.72	2002–2008	600k	17–89	Not reported	69%
**Eastern Europe**						
Czech Republic	1.12	1994–2000	10 million	5–75	30	68%
Czech Republic	1.16	1994–2011	10.3 million	5–75	33	69%
Estonia	1.4	2001–2010	1.3 million	Not reported	39.9	64%
**Asia**						
Japan	4.2	2009–2010	128 million	9–65	39	50%
Singapore	1.8	1976–2008	4.8 million	Not reported	Not reported	Not reported
**Australia**						
Australia	10.5	1995–1997	4.5 million	4–75	Not reported	66%
**South America**						
Peru	1.7	1985–1995	6.7 million	Not reported	Not reported	Not reported
Brazil	0.33	1993–2007	186 million	19–60	35	49%
**Africa**						
South Africa	0.06	2000–2009	3.5 million	40–60	36.8	Not reported

### Incidence of IgA Nephropathy over Time in the United States

The studies included in this review were conducted from 1974 to 2014. Three US studies reported incidence over time (Southern California, Minnesota, and Kentucky). In Olmsted County, the incidence of biopsy-proven IgA nephropathy rose from 0.7 per 100,000 for 1974–1983 to 0.9 per 100,000 people for 1984–1993 and 2.1 for 1993–2003.^19^ In Southern California, incidence increased from 0.1 to 1.6 per 100,000 from 2000 to 2011.^17^ In Central and Eastern Kentucky, incidence increased from 0.5 per 100,000 in 1975–1979 to 12.4 per 100,000 in 1990–1994 (Table 1).^16^ However, the incidence of IgA nephropathy varied more among regions than over time.

### Incidence of IgA Nephropathy by Sex in the United States in the Context of International Studies

**Figure 5.**
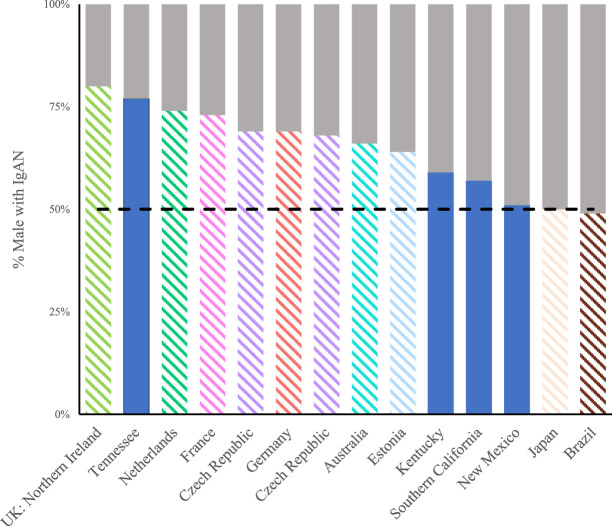
**Proportion of male patients with incident IgA nephropathy in US and international studies.** Male patients are represented as colored bars and female patients represented as gray bars within US and international studies reporting the proportion of IgA nephropathy–positive biopsies on the basis of sex. Only studies reporting sex data were included in this figure.

Four US and ten international studies reported sex-specific IgA nephropathy incidence or data (Figure 5). In most studies, except for Japan and Brazil, IgA nephropathy was over-represented in male patients compared with female patients. The proportion of male patients among those with IgA nephropathy ranged from 70% in Northern Ireland to 49% in Brazil, with a median of 67%.^24,25^ Of the four US study samples, only Tennessee had more than 50% male patients.^18^

This systematic review of the epidemiology of IgA nephropathy found a 3.5-fold variability in reported IgA nephropathy incidence across US studies and a >100-fold variability internationally. The incidence of IgA nephropathy is generally believed to be higher in White patients than in Black patients; this result was observed in all the US studies with the exception of a single study from Tennessee that was conducted within a much smaller pediatric cohort.^14,26^ However, the overall evidence for race-based differences in IgA nephropathy was sparse. In the future, larger population-based studies may help to determine the true incidence of IgA nephropathy and its association with race and other factors. Ideally, such studies would be longitudinal, would be conducted in diverse background populations, would use a unified classification system for IgA nephropathy, and would have similar access to kidney biopsy among those with suspected IgA nephropathy regardless of race.^27^

None of the five US populations studied in this systematic review exactly reflect the racial/ethnic distribution of the United States. The Minnesota and Kentucky studies were conducted in nearly all-White populations. The Tennessee study was small and focused only on children, an important consideration because kidney biopsy is more selective among children compared with adults. The New Mexico study over-represented Native Americans and Hispanics.^7, 16–19^.

Our review did not find strong data to support the notion that IgA nephropathy incidence is lower in Black patients than in White patients in the United States. Of the three US studies that examined IgA nephropathy incidence by race (in Tennessee, Kentucky, and California), only the California study found lower incidence in Black patients (0.1 per 100,000) than in White patients (0.72 per 100,000).^17^ That study was the largest of the three studies and conducted among adults (ages 18–84 years) who were mostly employed and insured, with good access to health care and to kidney biopsy if providers deemed it appropriate. By contrast, the Tennessee study found a higher incidence of IgA nephropathy in Black patients (0.57 per 100,000) than in White patients (0.3 per 100,000), but this study was based on only 17 pediatric cases.^18^ These two studies suggest that different referral and biopsy patterns may lead to differences in measured IgA nephropathy incidence^18^; however, the differences between these studies make them hard to compare.

Individuals of Hispanic ethnicity represent a large and growing segment of the US population (18.7% in 2020), yet they have received little attention for IgA nephropathy.^13^ Only one US study (from California) provided specific IgA nephropathy incidence data for individuals self-identified as Hispanic.^17^ In this study, the incidence of IgA nephropathy in Hispanic individuals was intermediate between the incidence in White (lowest) and Asian (highest) individuals. More data are needed about IgA nephropathy in Hispanics, who are projected to comprise 28% of the US population by 2060.^28^

Outside the United States, IgA nephropathy incidence is known to vary among countries and regions, even in geographically close populations, such as those in Eastern Europe, with similar ethnic backgrounds.^29^ This variability may be attributable to differences in biopsy practices. Of note, in some countries, such as Japan, urinalysis is more common than it is elsewhere, leading to more kidney biopsies and in turn to more IgA nephropathy diagnoses.^14,30^

Feehally and Barratt observed that IgA nephropathy incidence differs between male patients and female patients in European studies, but less so in Asian studies.^14, 29–31^ All the studies we reviewed (except those in Brazil and Japan) found the incidence of IgA nephropathy to be higher in male patients than in female patients.^30^

We tried to find studies of IgA nephropathy incidence in other countries and regions, especially Africa, in part for insight into the association of race/ethnicity with IgA nephropathy. However, only one Africa-based study (South Africa) met our criteria for inclusion. The IgA nephropathy incidence it reported was very low (0.06 per 100,000) and not stratified by race. The South Africa study was conducted in Cape Town, which has a relatively large White population (16% in 2021).^32^ However, the study sample was 42% Black, 53% mixed race, and 4% White, suggesting a sampling bias that may be related to biopsy availability^9^ and the racial distribution of the patients at the Groote Schuur Hospital, where the study was conducted.^32^ Studies of IgA nephropathy in African populations are sorely needed, and future studies of IgA nephropathy incidence in Africa should present data on the underlying local population demographics, as well as the demographics of the patients who undergo kidney biopsy.

A prior analysis of the geographic distribution of IgA nephropathy incidence on the basis of gene loci implied that ancestry was important in IgA nephropathy risk.^26^ Risk alleles associated with IgA nephropathy varied in an East to West and South to North orientation, meaning that Asian populations had more risk alleles than European populations, which in turn had more risk alleles than those of African ancestry.^26^ The geospatial analysis suggested a strong north to south incidence gradient; northern Europeans (*e.g.* Orkney Scots and those from Northwestern Russia) had higher IgA nephropathy incidence than populations from more southern areas within the same nations.^26^ Genomic data can help to parse out regional genetic differences that may exist, and that US studies can blur, but such studies are resource-intensive, and enrollment of diverse populations (*e.g.*, Hispanics) is needed to accurately describe the US population. Again, access to biopsy has the potential to bias genomic data.

Our study has some strengths. To the best of our knowledge, it is the first systematic review to focus on the epidemiology of IgA nephropathy and to examine its racial/ethnic variability. We included all relevant US studies and placed them in context by retrieving data related to the background population from alternative sources such as the US census and by examining international studies. However, our data had limitations. Most notably, we found only three US studies that reported specific incidence rates of IgA nephropathy by race and ethnicity and only one Africa-based study. That study took place in South Africa, which has a large White population. Therefore, our ability to address racial/ethnic differences in IgA nephropathy incidence was limited. The data generated were underpowered, and we did not attempt statistical testing.

In sum, this systematic review of the incidence of IgA nephropathy across populations supported the hypothesis that the true incidence of IgA nephropathy is higher in Asians than in non-Asians and in male patients than in female patients. The overall incidence of IgA nephropathy ranged very widely: 3.5-fold among US studies and >100-fold among international studies. Future studies of the epidemiology of IgA nephropathy should report data stratified by race/ethnicity and include both demographic data for the background population and demographic data for those who undergo kidney biopsy.
